# Nanomechanical electro-optical modulator based on atomic heterostructures

**DOI:** 10.1038/ncomms13590

**Published:** 2016-11-22

**Authors:** P. A. Thomas, O. P. Marshall, F. J. Rodriguez, G. H. Auton, V. G. Kravets, D. Kundys, Y. Su, A. N. Grigorenko

**Affiliations:** 1 School of Physics and Astronomy, University of Manchester, Manchester M13 9PL, UK; 2 School of Computer Science, University of Manchester, Manchester M13 9PL, UK

## Abstract

Two-dimensional atomic heterostructures combined with metallic nanostructures allow one to realize strong light–matter interactions. Metallic nanostructures possess plasmonic resonances that can be modulated by graphene gating. In particular, spectrally narrow plasmon resonances potentially allow for very high graphene-enabled modulation depth. However, the modulation depths achieved with this approach have so far been low and the modulation wavelength range limited. Here we demonstrate a device in which a graphene/hexagonal boron nitride heterostructure is suspended over a gold nanostripe array. A gate voltage across these devices alters the location of the two-dimensional crystals, creating strong optical modulation of its reflection spectra at multiple wavelengths: in ultraviolet Fabry–Perot resonances, in visible and near-infrared diffraction-coupled plasmonic resonances and in the mid-infrared range of hexagonal boron nitride's upper Reststrahlen band. Devices can be extremely subwavelength in thickness and exhibit compact and truly broadband modulation of optical signals using heterostructures of two-dimensional materials.

## Abstract (web summary)

Van der Waals heterostructures can be combined with metallic nanostructures to enable enhanced light–matter interaction. Here, the authors fabricate a broadband mechanical electro-optical modulator using a graphene/hexagonal boron nitride vertical heterojunction, suspended over a gold nanostripe array.

## Graphical abstract

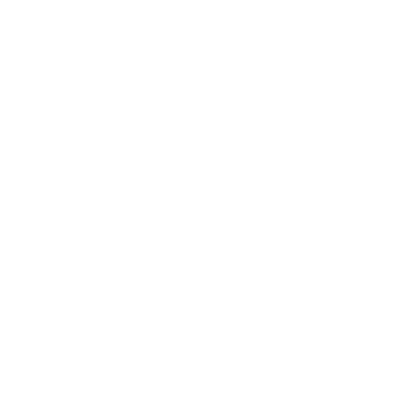

Plasmonics and the nano-optics of two-dimensional (2D) materials are two of the most rapidly advancing areas in photonics^1^,^2^,^3^. The advent of high-resolution nanofabrication techniques has allowed for the development of plasmonic nanostructures with a broad range of optical responses such as subwavelength confinement of light^4^ and ultra-narrow diffraction-coupled resonances^5^,^6^,^7^ that could lead to applications in waveguiding^8^ and biosensing^9^, respectively. Meanwhile, the extraordinary properties of 2D materials have generated great interest in the nano-optics community^10^. Graphene has demonstrated remarkable optical properties that include visual transparency defined by the fine structure constant^11^ and gate-tuneable intrinsic plasmons^3^,^12^,^13^. More recently, graphene has been combined with other 2D materials such as hexagonal boron nitride (hBN) to form heterostructures^14^ that have already been used to develop light-emitting diodes^15^.

The combination of plasmonic nanostructures with 2D heterostructures shows particular promise^3^. Placing plasmonic nanostructures in 2D heterostructure stacks allows for much stronger interactions between light and 2D materials^16^. This approach has been used to produce significant advances in the field of photovoltaics^17^, Raman spectroscopy^18^ and sensing^9^. Arguably the most exciting developments could come in optoelectronics^2^,^3^,^8^—the optical properties of graphene can easily be tuned by applying a gate voltage^19^. Combining graphene with metallic nanostructures capable of supporting ultra-narrow plasmon resonances could potentially lead to the development of compact optical modulators^3^. However, attempts to experimentally realize such a device have met with limited success^20^,^21^,^22^,^23^—modulation depths are typically small (or slow for graphene supercapacitors) and influence over higher-energy spectral features requires exceptionally high doping of graphene.

Here we use electromechanical modulation to achieve high modulation depths in a larger frequency range. Indeed, relatively little attention has been paid to the potential for nanoelectromechanical optical effects in either plasmonic or 2D heterostructure systems^24^,^25^. We describe a graphene/hBN/gold nanostripe heterostructure with an air gap between the hBN and plasmonic nanostripes. Application of a gate voltage between graphene and the gold nanostripe sublayer leads to a reduction in the air gap height. This in turn changes the effective electric field acting on graphene and hBN, modifying spectral reflection features from mid-ultraviolet to mid-infrared wavelengths. The demonstration of strong modulation (of up to 30%) in a 2D material-based device, particularly over such a broad range of spectral features, is unprecedented. It is important to stress that the volume in which the modulation is achieved is extremely small. For example, in the mid-infrared region (at a wavelength *λ* ∼7 μm) the optical interrogation volume is ∼*λ*^3^/10, the smallest reported so far—approximately three orders of magnitude smaller than reported in ref. 25. Our results open up new possibilities for strong light modulation in optoelectronic devices.

## Results

### Sample design

**Figure 1:**
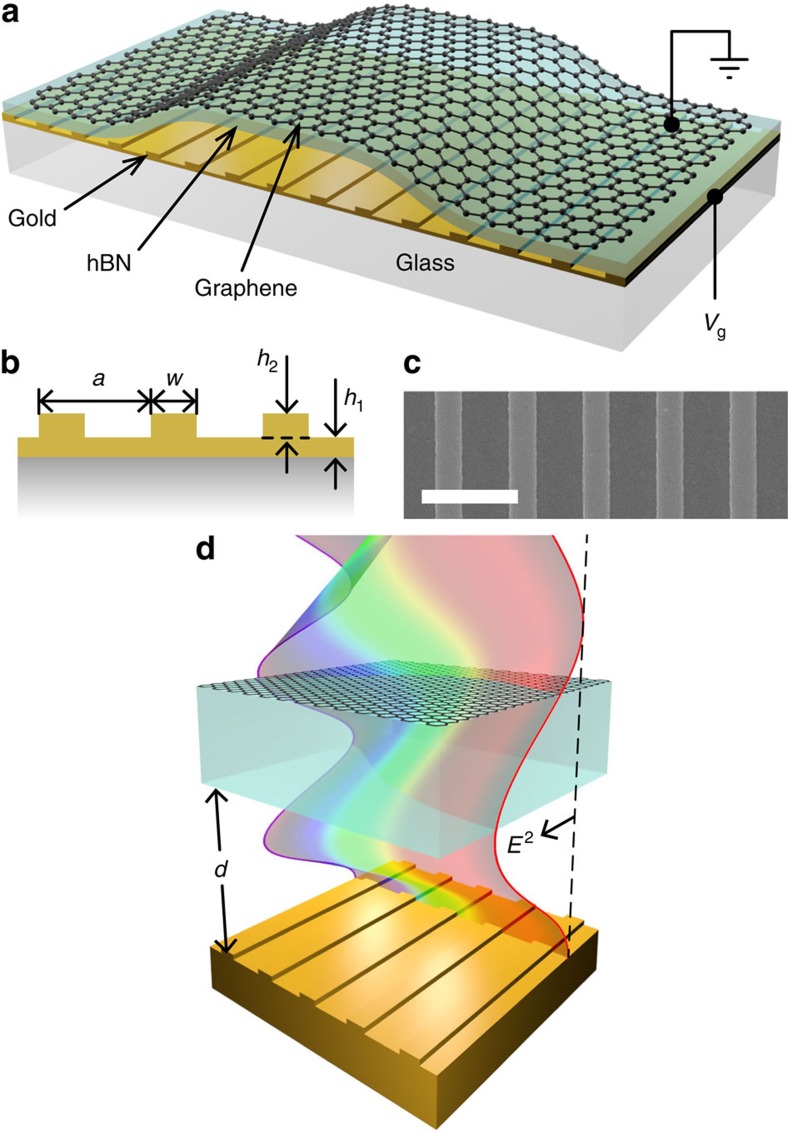
Nanomechanical electro-optical modulator structure. (**a**) Schematic of our device with air gap height *d*. (**b**) Geometric design parameters for our gold nanostripe array. (**c**) Representative scanning electron microscopy (SEM) micrograph of the nanostripe array (scale bar 2 μm). (**d**) The working principle of the device. The coloured wave represents an unperturbed standing wave for different wavelengths observed under reflection from the nanostructured mirror.

The design of our graphene/hBN/nanoarray modulator is shown in Fig. 1a. The gold plasmonic nanostripe array is separated from the graphene/hBN by an air gap (*d*). Using the graphene as a broadband, transparent and extremely tough electrical contact, a gate voltage can be applied across the structure. In this work the nanostripe array had a periodicity *a*=1,570 nm, stripe width *w*=550 nm, stripe height *h*_2_=80 nm and gold sublayer of thickness *h*_1_=65 nm (Fig. 1b)—see Methods for device fabrication details. A representative scanning electron microscopy micrograph is shown in Fig. 1c. Such plasmonic nanoarrays can be tuned to give narrow, diffraction-coupled resonances that arise when the wavelengths of diffracted light modes, running along the air–substrate boundary (known as Rayleigh cutoff wavelengths), are recaptured as electron oscillations in the plasmonic nanostructures^5^. These resonances can be further narrowed by adding a metallic sublayer^26^. Our nanostructure was designed to produce a narrow plasmon resonance around the telecom wavelength of *λ* ∼1.5 μm, although higher-order diffraction-coupled modes exist throughout the near-infrared and visible spectrum. An hBN flake (∼110 nm thick) and single-layered graphene were then mechanically exfoliated and transferred on to the plasmonic nanostructure (see Methods). Hexagonal boron nitride is an ideal dielectric for graphene devices because it is an atomically flat crystal with a similar lattice constant to graphene^27^. Within the modulator region of our device the initial air gap between the hBN and nanostripe array was ∼300 nm. An optical image of the completed device is shown in Supplementary Fig. 1. Atomic force microscopy data confirming the device dimensions is shown in Supplementary Fig. 2.

### Spectroscopic ellipsometry and reflectometry

**Figure 2:**
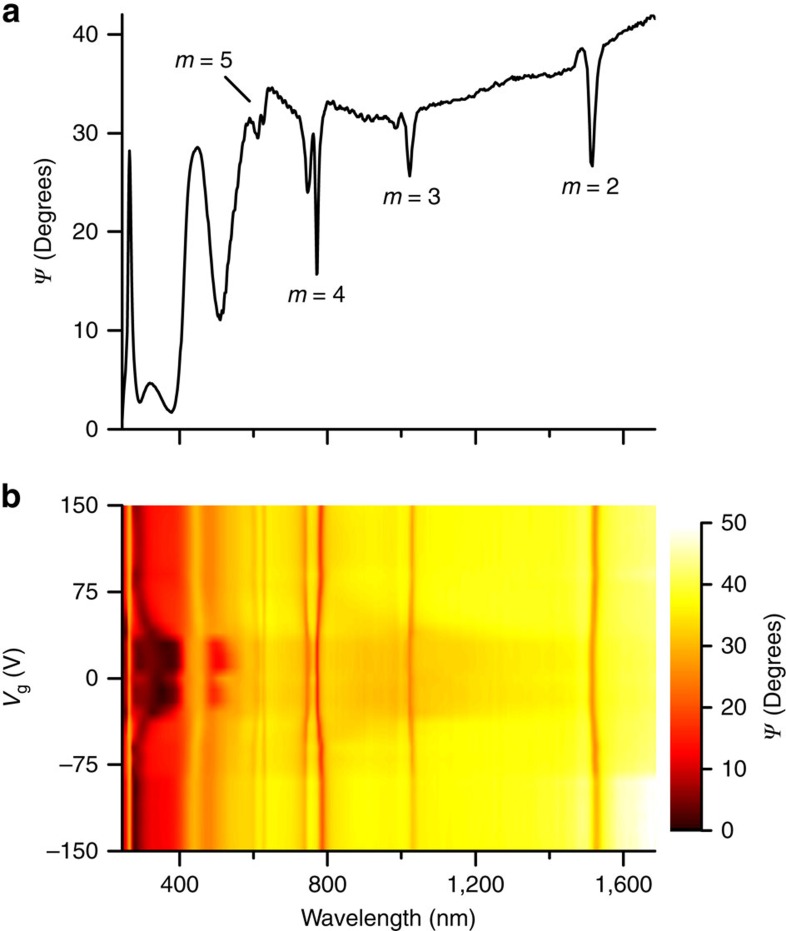
Device characterization using spectroscopic ellipsometry. (**a**) Ellipsometric reflection spectrum *Ψ* of our graphene/hBN/plasmonic heterostructure (*V*_g_=0 V, *θ* =70°). (**b**) Colour map of *Ψ* as a function of wavelength and *V*_g_.

Devices were studied using spectroscopic ellipsometry and reflectometry (see Methods). Figure 2a shows the ellipsometric reflection spectrum (*Ψ*) of a device from the mid-ultraviolet through to the near-infrared, when illuminated at an incident angle of *θ*=70°. We attribute the broad absorption peaks in the wavelength range 280 nm<*λ*<590 nm to Fabry–Perot (FP) interference in the air gap, whereas the sharp feature at *λ*=275 nm is caused by the complex, multi-peaked ultraviolet absorption spectrum of hBN^28^. The remaining strong features from 590 nm<*λ*<1,600 nm primarily arise from the nanoarray and correspond to the Rayleigh cutoff wavelengths for air, determined by 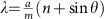, where *a* is the array periodicity, *m* is an integer and *n* the refractive index of air^5^. The absorption peaks at *λ*≈620, 780, 1,030 and 1,520 nm can be associated with the *m*=5, 4, 3 and 2 diffraction-coupled modes, respectively. The *m*=4, 5 features each consist of two peaks because of a mismatch between the inverse polarizability and retarded dipole sum of individual nanoparticles in the plasmonic nanoarray in this spectral region^29^, caused by the presence of the hBN.

Figure 2b plots *Ψ* as a function of both wavelength and gate voltage *V*_g_, showing how the various features from the ultraviolet to near-infrared respond to electrical biasing of the device. As *V*_g_ is raised from 0 to ±150 V we see dramatic changes in the FP resonances, along with a red-shift of the Rayleigh resonance wavelengths. These changes in reflection happen because of motion of the hBN/graphene heterostructure—the applied gate voltage creates an electrostatic force within the device, acting to reduce *d*. The Maxwell stresses caused by induced electrical charges can be of the order of 10 atmospheres (see Supplementary Note 1). We note that Casimir interactions between the graphene and gold are negligible at this length scale^30^. It is worth noting that, in general, the opto-electro-mechanical response of the structure is symmetric with respect to the sign of *V*_g_ and there is a threshold of ±50 V before which application of *V*_g_ produces no change in optical reflection (no motion of the heterostructure). One exception to the above mechanism occurs near *λ*=1.6 μm for large negative *V*_g_. In this region the absorption changes because of electrical gating of graphene, moving its charge neutrality point and the spectral onset of optical Pauli blocking^3^. This results in an increase in the measured value of *Ψ* at the highest wavelengths for the largest negative *V*_g_. It is worth noting that one can distinguish the effect of hBN motion and that of the Pauli blocking by the symmetry of the response (the effect of hBN motion is symmetric with respect to the sign of the applied voltage, whereas the Pauli blocking effect is not symmetric because of the initial doping of graphene) and by the wavelength range at which these effects are observed (see the discussion in Supplementary Note 2).

**Figure 3:**
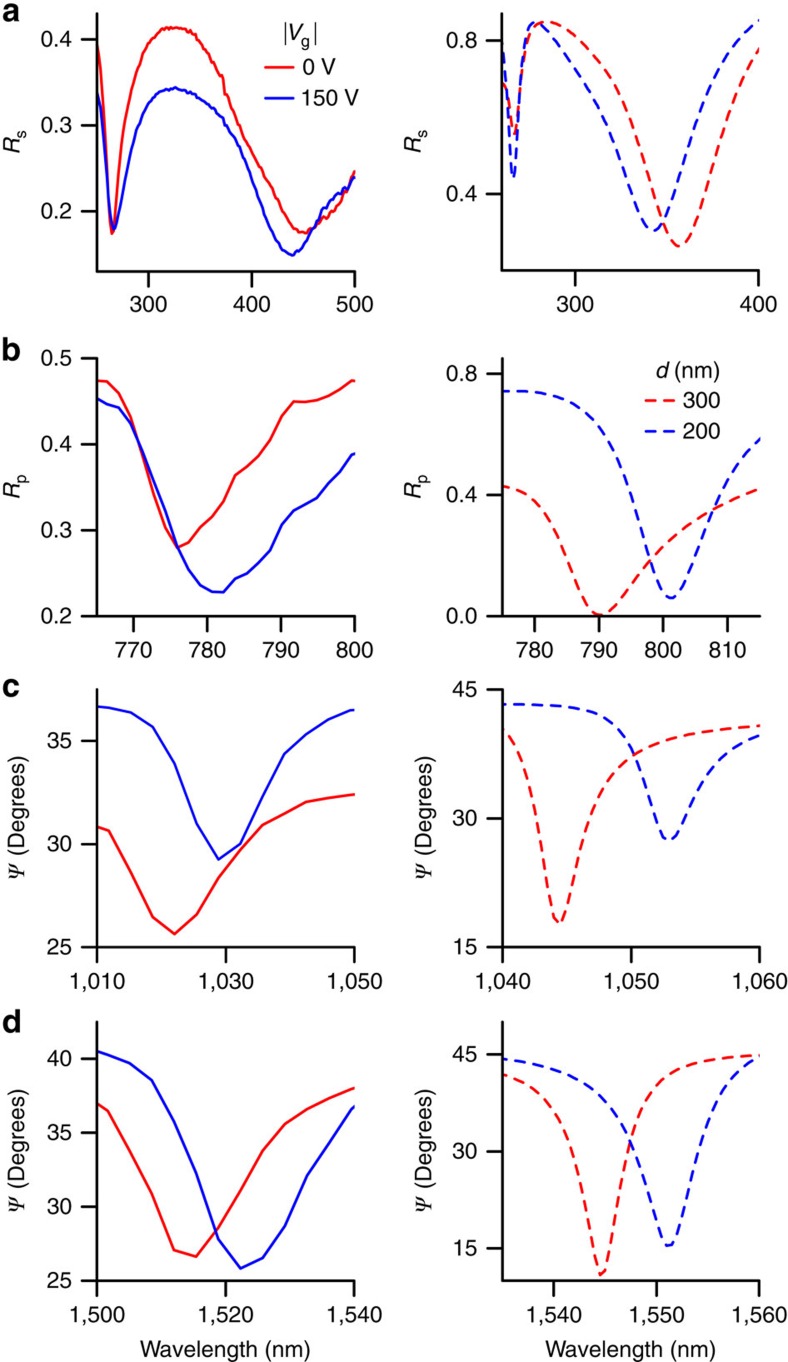
Electromechanical modulation from ultraviolet to near-infra red regions. Measured reflection spectra (left column) of the graphene/hBN/nanoarray for (**a**) *s*-polarized light (*R*_s_) in the ultraviolet/blue range and (**b**) *p*-polarized light (*R*_p_) in the visible. (**c**,**d**) Ellipsometric reflection in the near-infrared. All spectra were measured with *θ*=70°. Modelling results (right column) reveal that the electrically induced reflection modulation is explained by a changing air gap. The sharp reflection minima in (**a**) (centred at *λ*=275 nm) stems from the complex ultraviolet absorption spectrum of hBN. The features in (**b**–**d**) correspond to the fourth-, third- and second-order Rayleigh cutoff wavelengths of the nanoarray, respectively.

The response to *V*_g_ in the ultraviolet region measured in *s*-polarized reflection (*R*_s_) is shown in Fig. 3a. Note that the hBN absorption feature at *λ*=275 nm is relatively insensitive to *V*_g_ (and hence *d*) as the wavelength is approximately equal to the optical path length within the hBN. However, as *V*_g_ is increased (and *d* decreased) the adjacent FP absorption features are dramatically altered. This cavity modulation effect occurs over the entire measured ultraviolet range (*λ* >250 nm) and gives a peak modulation depth of ∼10% at *λ* ∼310 nm. This compares favourably with prior attempts at ultraviolet modulation that have usually relied on wide band gaps^31^ or excitonic effects^32^ in semiconductor devices.

Moving to the visible and near-infrared ranges (for the same device) we have observed strong modulation of the diffraction-coupled resonances in the plasmonic nanoarray produced by graphene gating. Figure 3b shows the changes in *p*-polarized reflection spectra for the *λ*=780 nm resonance, whereas Fig. 3c,d show *Ψ* of ∼1,030 and 1,520 nm respectively. In all three cases the reflection minima are shifted by up to 10 nm for |*V*_g_|=150 V—as the hBN moves closer to the nanoarray the local fields increase and the refractive index of the ambient medium (ordinarily *n*=1 for air) is effectively changed. This in itself produces a strong modulation effect, simply because the plasmonic resonances are so narrow. Even though the minimum *Ψ* value in the vicinity of *λ*=1,520 nm (Fig. 3d) remains approximately constant (∼26–27°), its shifting wavelength with increasing |*V*_g_| leads to a modulation depth of 20% at *λ*=1,536 nm. This is significantly higher than the near-infrared absolute modulation depths previously reported in graphene- and plasmonic-based devices^23^,^33^,^34^,^35^: for example, Li *et al*.^35^ reported an absolute modulation depth of ∼9% at telecom wavelengths using a graphene-clad microfibre. The modulation frequency in our devices could be reasonably high ∼100 kHz and could be further improved to 100 MHz by a dedicated design (see the detailed discussions in Supplementary Notes 3 and 4).

Reflection spectra obtained experimentally are described well by rigorous coupled-wave analysis (RCWA) modelling (see Methods), in which the graphene/hBN heterostructure is displaced vertically, reducing *d* from 300 to 200 nm. The right-hand panels in Fig. 3a–d show the results of this modelling in the ultraviolet, visible and near-infrared regions of interest. Both the functional form and behaviour of the blue-shifting FP and red-shifting Rayleigh resonances are reproduced by the model. The differences between the experimental and modelled spectra can be attributed to impurities in, and roughness of, our gold nanostripe arrays. In addition, the height of the suspended area is not constant over the beam spot that leads to resonances smearing. Further modelling, of even smaller air gaps, reveals that as *d* approaches zero the Rayleigh resonance wavelengths become increasingly sensitive to changes in *d* (see Supplementary Fig. 3). With this knowledge future devices might be deliberately engineered with smaller initial air gaps, potentially greatly enhancing the achievable modulation depths.

**Figure 4:**
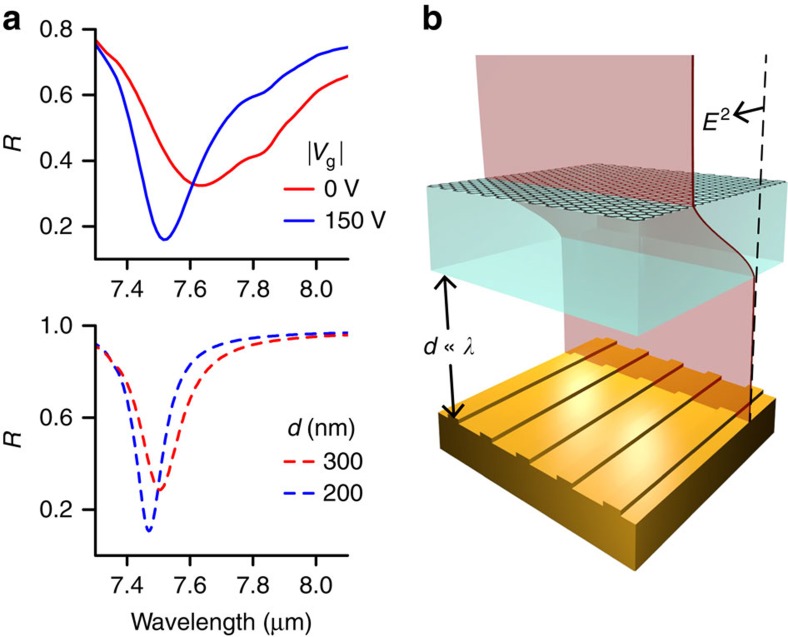
Modulation and modelling of hexagonal boron nitride's Reststrahlen band. (**a**) Measured (upper panel) and modelled (lower panel) reflection spectra close to the transverse optical (TO) phonon energy in hBN. The broader measured resonance width stems from the Fourier transform infrared (FTIR) reflecting objective that provides a range of incidence/measurement angles simultaneously (*θ*=12–24°) compared with the modelled *θ*=15°. (**b**) The air gap is significantly smaller than the mid-IR wavelengths; however, close to the TO phonon energy, light is highly compressed within the hBN because of its large index of refraction.

Infrared reflection spectra were measured using Fourier transform infrared spectroscopy (see Methods). Figure 4a shows the strong modulation feature observed in the mid-infrared wavelength range produced by graphene gating. At such long wavelengths, incident light is not influenced by the grating structure, instead effectively experiencing a planar gold surface. On the long wavelength side of its Reststrahlen band, just beyond the transverse optic phonon wavelength of ∼7.35 μm, hBN displays strong light absorption and spectral dispersion. In this region we observe a broad reflection minimum, with reflection values falling to 33% at *λ* ∼7.6 μm (Fig. 4a). The electromechanical reduction of *d* induces a narrowing of this feature and a blue shift of the reflection minimum by over 100 nm. The reflection minimum also falls to ∼15%. As a result, large absolute reflection modulation depths are possible for a given wavelength—up to 30% at *λ*=7.5 μm. This represents a dramatic improvement on existing mid-infrared graphene-plasmonic modulator results^22^,^36^,^37^: for example, Yao *et al*.^37^ have previously reported an absolute reflection modulation depth of ∼20% at ∼7.6 μm. Comparing the device dimensions (total height ∼450–550 nm) with the wavelength of this absorption feature reveals the high degree of light confinement within the structure. As a consequence of this confinement—on the order of ∼*λ*/10—the device is capable of modulating light with an optical interrogation volume of as little as *λ*^3^/10. (The actual modulation volume of our device was ∼*λ*^3^ at mid-infrared frequencies. It can be reduced to the optical interrogation volume by using smaller suspended areas.)

As with shorter wavelengths, RCWA simulations agree very closely with the measured mid-infrared behaviour (Fig. 4a). The basic existence of the absorption feature at *λ* ∼7.5 μm is attributable to material absorption in hBN. Hence, a similar reflection minimum also occurs in simulations of free-standing thin hBN films, and could be modulated simply by changing the hBN thickness (for example, from 110 to 140 nm). However, the hBN thickness is fixed experimentally. Instead, we observe an analogous modulation effect arising from the motion of the hBN slab with respect to the gold mirror. Altering *d* leads to changes in the optical field overlap with the hBN, as the field diminishes to zero at the gold surface (Fig. 4b). Although the nanostripe array was included in the above RCWA calculations, a much simpler Fresnel reflection-based model of a planar Au/air/hBN/graphene structure confirmed that it is not necessary for the observed mid-infrared modulation.

## Discussion

One potential application of this modulation technique is the study of the nanomechanics of suspended 2D materials. Our modulator design could be adapted to detect the motion of a 2D heterostructure by measuring the dispersive coupling strength *g*=Δ*E*/Δ*z*, where Δ*E* is the shift in the collective plasmon energy and Δ*z* the displacement of the membrane. In the case of a plasmonic nanoarray supporting diffraction-coupled plasmon resonances with the 2D heterostructure suspended close to its surface, this number was *g*=4 meV nm^–1^ or 1,000 GHz nm^–1^ that is quite large for a plasmonic device.

In conclusion, we have shown that introducing an air gap of variable height into a graphene/hBN/nanoarray structure can allow for very strong optical reflection modulation via graphene gating. We have demonstrated that this effect can be used to create modulation from the mid-ultraviolet all the way to the upper Reststrahlen band of hBN (mid-infrared). The use of 2D atomic heterostructures allows for an extremely small optical interrogation volume of ∼*λ*^3^/10. To see such strong effects over such a broad spectral range in a device controlled by graphene gating is unprecedented and opens up many exciting possibilities in the development of optical nanoelectromechanical systems.

## Methods

### Nanoarray fabrication

Uniform arrays of gold nanostripes (total area=300 × 100 μm) were fabricated (with a gold sublayer) on glass substrates using standard electron beam lithography and electron beam evaporation techniques^9^,^26^. First, the glass substrate was covered with a thin film of Cr (3 nm) to prevent charging during lithography, followed a flat region of Cr (3 nm, for adhesion) and Au (65 nm). As well as forming the metallic sublayer, this region acted as the back contact for electrical measurements. Next, the array of gold nanostripes (length 100 μm) was fabricated on top of the gold sublayer, once again using a Cr (3 nm) adhesion layer. Finally, the exposed areas of the initial Cr film were removed via wet chemical etching. Nanostripe dimensions were confirmed using scanning electron microscopy.

### Graphene and boron nitride transfer

Single-layered graphene and hBN were prepared by standard micromechanical exfoliation^1^. hBN was obtained from the Advanced Materials Laboratory, National Institute for Materials Science, Japan. The hBN and graphene flakes (both ∼50 × 100 μm) were deposited onto separate oxidized Si wafers. Each flake was then delaminated by spinning a layer of poly(methyl methacrylate) on top of the substrate and etching away the oxide layer. Sequential flake transfers to the nanoarray were performed using optical microscope-mounted micromanipulator. After each transfer the poly(methyl methacrylate) membrane was dissolved with acetone. Finally, the graphene/hBN/gold nanoarray was annealed in an Ar/H_2_ gas mixture at 275 °C for 3 h. Adhesion between the hBN and rough nanoarray surface was non-uniform over the flake area, leading to the existence of the required air gap in regions of each device. The nanostripe array period is significantly larger than width of the individual nanostripes, resulting in a small contact area between the hBN and nanoarray surface. The gaps in physical contact have a natural tendency to propagate, leading to a high probability of the formation of air gaps (bubbles) during heterostructure transfer.

### Optical and electrical measurements

Ellipsometric and reflection spectra were measured in a Woollam M-2000F focussed beam ellipsometer (Numerical Aperture 0.1). At the highest angles of incidence (∼80°), with the strongest localized plasmon resonances), the measurement spot size was larger than the graphene/hBN area; the angle of incidence was reduced to 70° to ensure the measurement spot was incident only on the active device area. Ellipsometry characterizes a sample's optical properties via the ellipsometric parameters *Ψ* (ellipsometric reflection) and *Δ* (ellipsometric phase shift) that are related by the equation tan(*Ψ*)exp(*iΔ*)=*r*_p_/*r*_s_, where *r*_p_ and *r*_s_ are the amplitude reflection coefficients for *p*- and *s*-polarized light, respectively. Ellipsometry measures the ratio of reflection coefficients, making it immune to many sources of noise and therefore a highly sensitive technique. Fourier transform infrared spectroscopy was performed using a Bruker Vertex 80 system with a Hyperion 3000 microscope. A variety of sources and detectors combined with aluminium-coated reflective optics enable this system to be used from visible to THz wavelengths. A reflecting objective provides a range of incident angles (*θ*=12–24°) and the entire beam path purged with dry, CO_2_-scrubbed air to minimize strong atmospheric infrared absorption bands. Gate voltages were applied using a Keithley 2400 Sourcemeter. All measurements were performed under standard room temperature and pressure conditions.

### Modelling

Simulations were performed using the Reticolo package in Matlab^38^ that relies upon a RCWA method^39^. The optical properties of Au were calculated within this package using the Lorentz-Drude model and the dispersion relation of hBN was calculated using the Lorentz oscillator model^40^


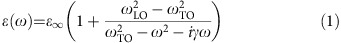


where *ω*_LO_ and *ω*_TO_ are the longitudinal optical and transverse optical phonon frequencies respectively, *ɛ*_*∞*_ is the high frequency permittivity and *γ* is the damping constant associated with the plasma oscillation. An additional spectral absorption feature was introduced to the modelled hBN in order to reproduce the experimentally observed absorption peak in the near-ultraviolet region. Graphene optical conductivity was taken in random-phase approximation^12^.

### Data availability

The data that support the findings of this study are available from the corresponding author upon request.

## Additional information

**How to cite this article:** Thomas, P. A. *et al*. Nanomechanical electro-optical modulator based on atomic heterostructures. *Nat. Commun.* 7:13590 doi: 10.1038/ncomms13590 (2016).

**Publisher's note**: Springer Nature remains neutral with regard to jurisdictional claims in published maps and institutional affiliations.

## Supplementary Material

Supplementary Information Supplementary Figures 1-8, Supplementary Notes 1-4, Supplementary References
