# Supplementary material for: Nanomechanical electro-optical modulator based on atomic heterostructures
Source: Nat Commun. 2016 Nov 22;7:13590. doi: 10.1038/ncomms13590 (PMC5121424; doi:10.1038/ncomms13590)
Supplement: Supplementary Information — Supplementary Figures 1-8, Supplementary Notes 1-4, Supplementary References [file ncomms13590-s1.pdf]

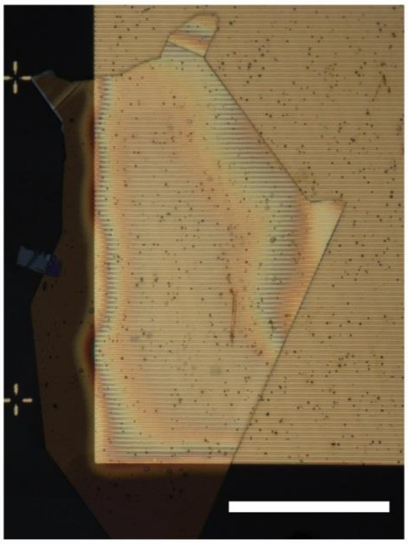

Supplementary Figure 1 | Optical image of graphene/hBN stack on gold nanostripe array. Scale bar 50 µm. Interference fringes indicative of an air gap are clearly visible. The presence of single layered graphene over the entire hBN area was confirmed with Raman spectroscopy.

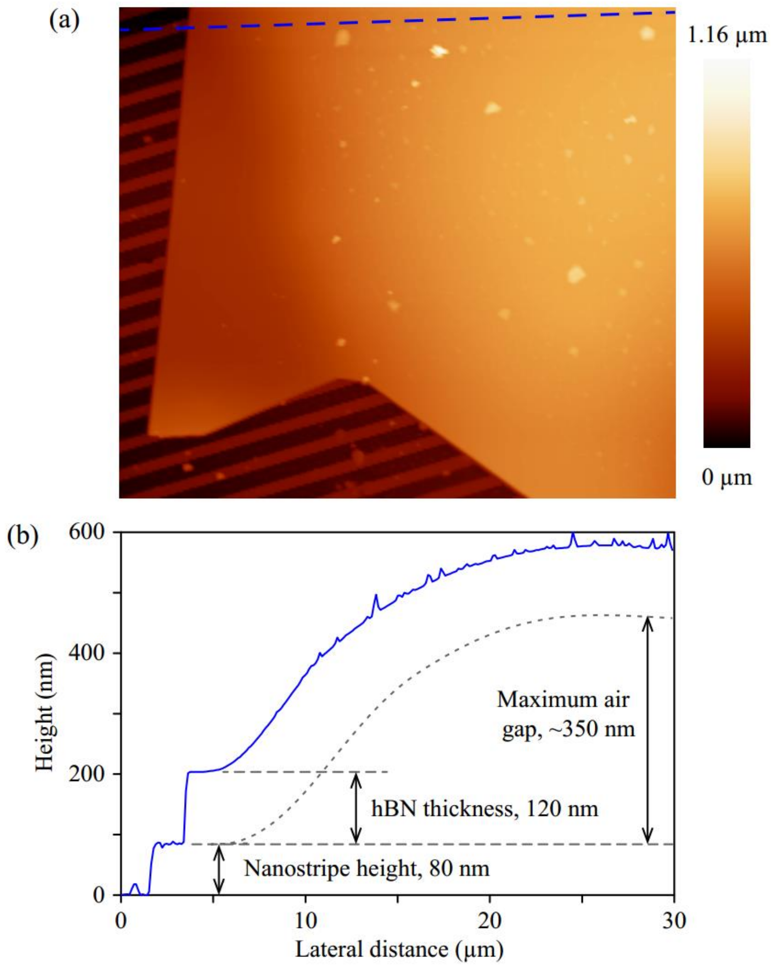

Supplementary Figure 2 | Atomic force microscopy (AFM) of graphene/hBN stack on gold nanostripe array. (a) AFM image of edge region of the graphene/hBN stack. (b) Profile along dashed line in (a). The purple circles show the theoretical fit for a clamped thin membrane.

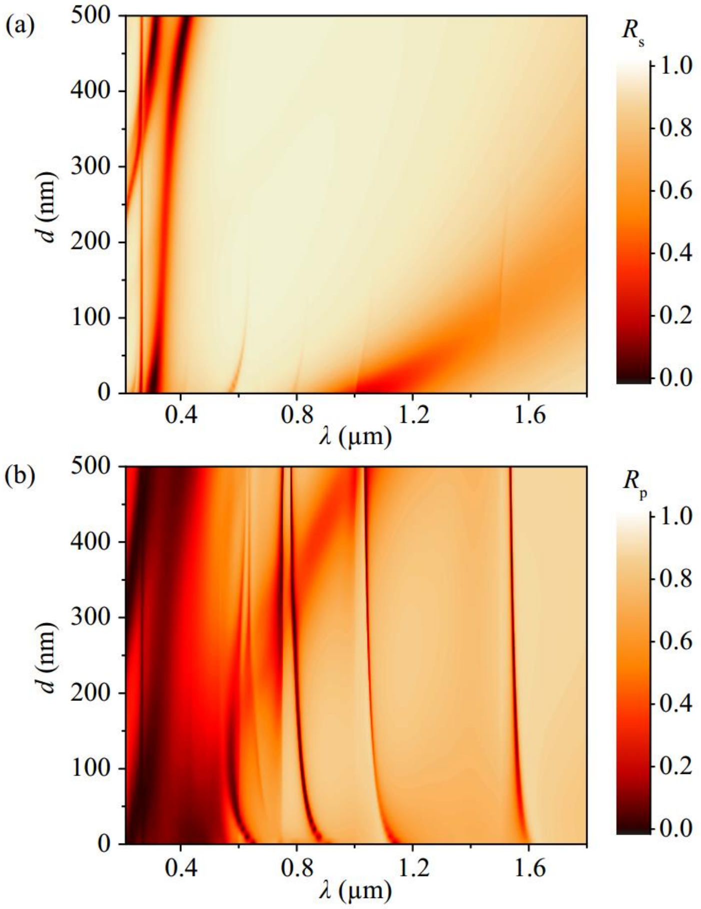

Supplementary Figure 3 | Modelled spectral reflection in the *λ*-*d* plane. Reflection for **a** s-polarized and **b** p-polarized incident light. Angle of incidence θ = 70°. For d < 50 nm the system becomes extremely sensitive to changes in d, leading to dramatically increased modulation depths.

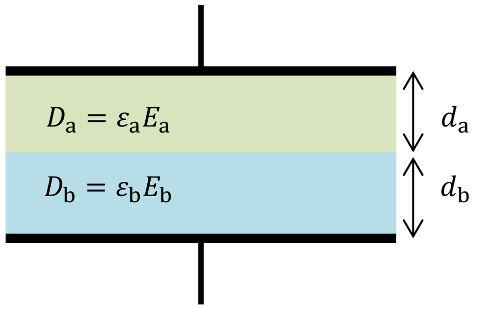

Supplementary Figure 4 | Model of electro-mechanical modulator as a parallel-plate capacitor. $D_{a,b}$, $\varepsilon_{a,b}$, $E_{a,b}$ and $d_{a,b}$ are respectively the displacement field, relative permittivity, electric field and thickness of the air gap (a) and the hBN (b).

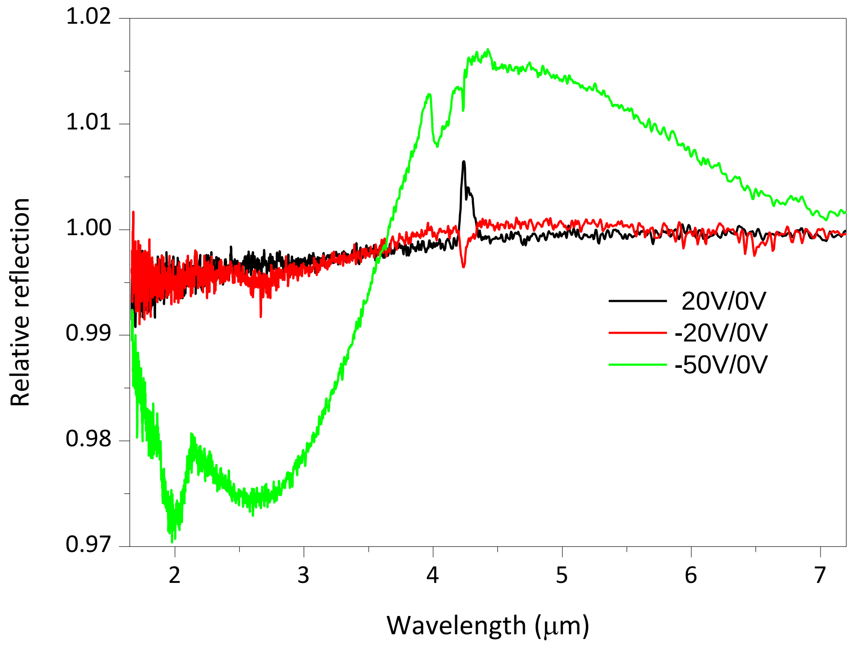

Supplementary Figure 5 | Relative reflection showing effect of Pauli blocking in graphene. Relative reflection of our device in the wavelength range 2-7 μm for V_g = ± 20 V, – 50 V.

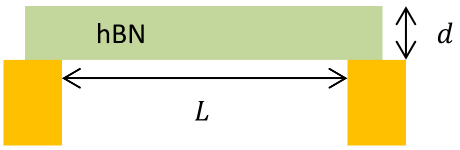

Supplementary Figure 6 | Model of electro-mechanical modulator as vibrating suspended hBN slab. *L* is the length of unfixed hBN and *d* is its thickness.

(a)

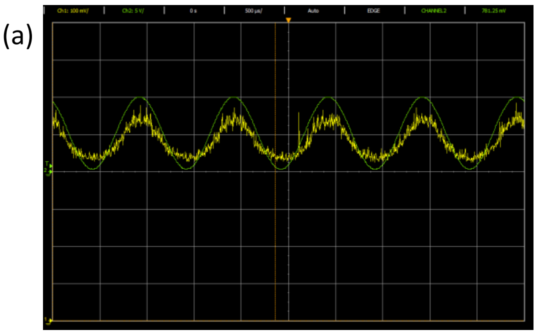

(b)

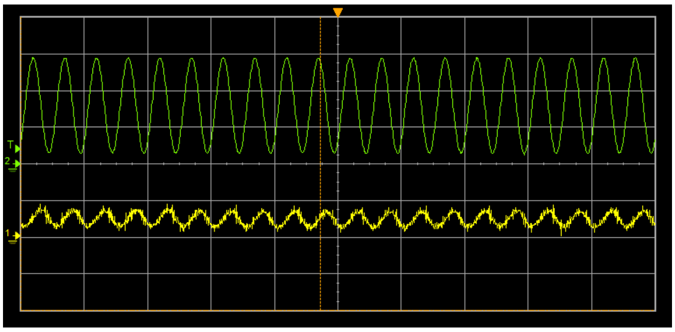

Supplementary Figure 7 | Oscilloscope traces showing operation of modulator. Example of the operation of our modulator in reflection measured with a laser with wavelength 1.06 µm. The modulation frequency was (a) 1 kHz and (b) 4 MHz. The green oscilloscope trace shows the applied gating voltage and the yellow trace shows the modulated optical signal measured with a photodiode. In (a) the modulation is shown including the dc component of the reflected light, with origin at the bottom of the graph. In (b), the lower modulation amplitude necessitated the use of ac coupling in the oscilloscope.

(a)

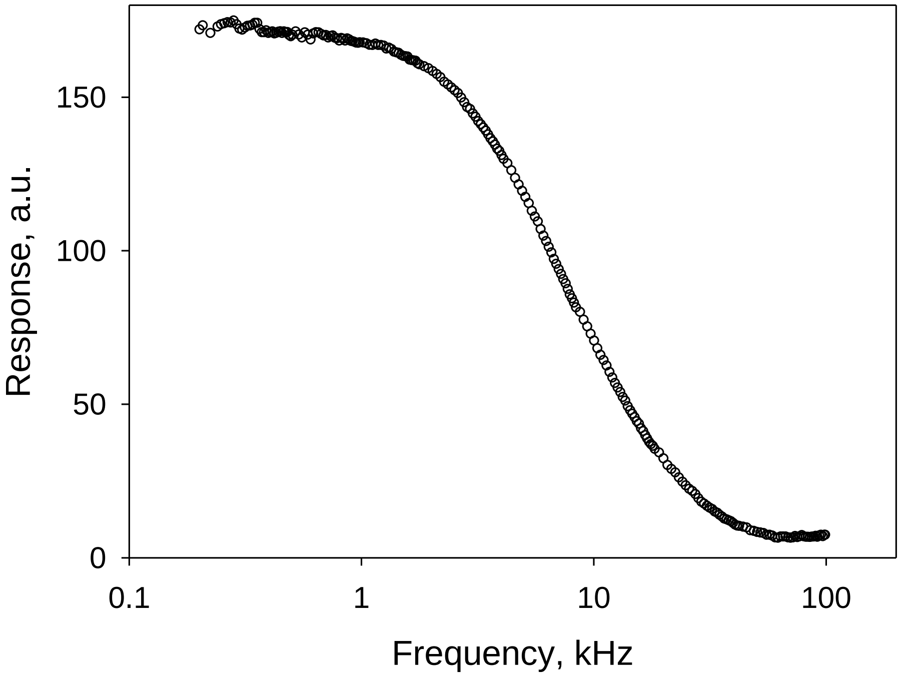

(b)

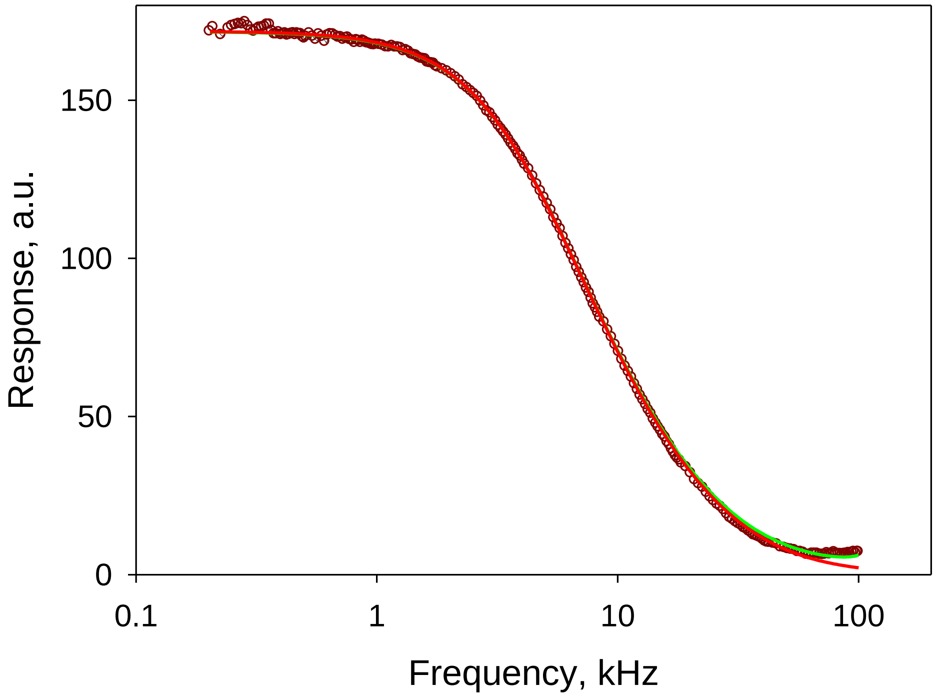

Supplementary Figure 8 | Frequency dependence of modulation. (a) Typical measured frequency dependence of the optical modulation amplitude in our modulator. (b) The same frequency dependence plot as in Supplementary Figure 8a but with a fit to an overdamped response (red curve) and a fit as the product of a harmonic oscillator response and the overdamped electrical response (green curve).

Supplementary Note 1 | Maxwell stresses

By treating our device as a parallel plate capacitor (with the graphene and gold nanoarray acting as the parallel plates, see Supplementary Figure 4), we estimate the Maxwell stresses experienced by the hBN and air within the device.

$D_{\mathrm{a,b}}$, $\varepsilon_{\mathrm{a,b}}$, $E_{\mathrm{a,b}}$ and $d_{\mathrm{a,b}}$ are respectively the displacement field, relative permittivity, electric field and thickness of the air gap (a) and the hBN (b) as defined in Supplementary Figure 4. We shall use $\varepsilon_{\mathrm{a}} = 1$, $\varepsilon_{\mathrm{b}} \approx 4$, $d_{\mathrm{a}} \approx 100$ nm (when the applied voltage $V = 150$ V), and $d_{\mathrm{b}} \approx 110$ nm. If the displacement field across the device is constant, then $D_{\mathrm{a}} = D_{\mathrm{b}}$ and $E_{\mathrm{a}} = \frac{\varepsilon_{\mathrm{b}}}{\varepsilon_{\mathrm{a}}} E_{\mathrm{b}}$. By finding the potential difference V across the device it can be shown that

$$E_{\mathrm{b}} = \frac{V}{d_{\mathrm{b}} + \frac{\varepsilon_{\mathrm{b}}}{\varepsilon_{\mathrm{a}}} d_{\mathrm{a}}}. \tag{1}$$

An equivalent expression can be found for E_{a}. We can then use this value to calculate the Maxwell stress σ experienced by each dielectric[1],

$$\sigma_{\mathrm{a,b}} = \varepsilon_0 \varepsilon_{\mathrm{a,b}} E^2, \tag{2}$$

where $\varepsilon_0 = 8.854 \times 10^{-12}$ F/m is the permittivity of free space. This yields a Maxwell pressure of around 20 atm (atmospheres) at the air-hBN interface at gating voltage of 100 V. The Grüneisen parameter is close to zero for most phonon modes in hBN, including the transverse optic phonon mode shown in figure 4, see Ref. [2], meaning that the modulation of the absorption feature at ~7.35 µm can only be attributed to the movement of the hBN flake.

Supplementary Note 2 | Optical Pauli blocking and the Fermi energy

In the modelling we have used values for the Fermi energy calculated with the help of a simple capacitance model. In order to check these calculations we have measured gated optical spectra of our devices at low gate voltages such that the onset of motion in the graphene/hBN stack was not yet reached due to mechanical hysteresis. As a consequence, the optical reflection of the device only changes due to the effect of optical Pauli blocking.

Supplementary Figure 5 shows the spectral dependence of relative reflection of our device in the wavelength range 2-7 μm (the relative reflection was measured as $R(V_g)/R(0)$). The black and red curves show the relative spectra at V_g = 20 V and V_g = –20 V. Since the initial doping of our exfoliated graphene was high, these spectral curves are close to unity, which is characteristic of the forbidden interband transition (and negligible intraband contribution). However, at V_g = –50 V we see change in the reflection centred around twice the Fermi energy position (at a wavelength of 3.8 μm) which is in a good agreement with a simple capacitance model for the Pauli Blocking (provided an initial doping is taken into account).

Supplementary Note 3 | Modulation frequency and amplitude

To estimate the maximum modulation frequency of our device we approximate our device as a slab of hBN vibrating in one dimension with both ends fixed, see Supplementary Figure 6.

In our case $L \approx 50$ μm is the length of unfixed hBN that is free to vibrate and $d \approx 110$ nm is the thickness of the hBN. We shall take the hBN width b (going into the page) to be approximately equal to L. Euler-Bernoulli beam theory gives the fundamental frequency f of the simply supported hBN plate as[3]

$$f = \frac{\pi d}{2L^2}\sqrt{\frac{E}{12\rho}}, \tag{3}$$

where $E \approx 70$ GPa is the Young's modulus of the hBN and $\rho = 2.2$ g cm^{-3} is the density of hBN. This gives a fundamental vibration frequency (and hence possible modulation frequency) of the order of 100 kHz, which is comparable with the upper limits of other recently reported nanomechanical modulators[4]. It is worth noting that the dedicated design (for example using $L \approx 5$ μm and smaller hBN thickness) would allow one to increase this frequency to around 100 MHz.

Supplementary Figure 1 provides an optical image of the graphene/hBN stack on gold nanostripe array studied in our experiments. We can see that the area of the suspended stack is approximately rectangular, although the clamped edges of the stack have a more complicated form. To assess the height profile of the suspended stack we performed AFM measurements, as shown in Supplementary Figure 2. Supplementary Figure 2a gives the AFM image of a sub-section of the device area, with Supplementary Figure 2b showing the height line-scan along the dashed line in Supplementary Figure 2a. Along the length of this line-scan we can clearly resolve the individual gold stripes in the uncovered region, as well as the area of hBN in contact with the gold stripes and the suspended region. The maximum measured air gap was around 350 nm, which is in good agreement with estimates we obtained from optical spectra fitting (see Figure 3 of the manuscript).

The purple circles in Supplementary Figure 2b show the theoretical fitting of the hBN profile using a simple model of a clamped thin rectangular membrane[3]. Since we have a good agreement between the calculated and the measured profile, we apply this simple model to quantitatively evaluate the change of the membrane height due to applied voltage. The maximal height of the clamped thin rectangular membrane with a smaller side (length *b*) and a larger side (length *a*) can be evaluated as $w = C \cdot (1 - v^2)\frac{(p_0 - p_\mathrm{a}) \cdot b^4}{E \cdot d^3}$, where $C = \frac{0.32}{1 + (b/a)^4}$, ν is the Poisson ratio, p_0 is the initial pressure inside the bubble, p_a is the atmospheric pressure, *E* is the Young modulus, and *d* is the membrane

thickness. In our case the length of the smaller side is around $b \cong 50$ μm, $b/a \cong 0.5$, $\nu \cong 0.3$ and $w \cong$ 350 nm, which yields the initial pressure inside the bubble $p_0 \cong 1.2$ atm. When the gate voltage is applied to graphene, the height of the membrane would decrease due to the increased top pressure by the electrostatic pressure p_e (evaluated in the section above) and counteracted by the increased pressure inside the bubble $\tilde{p}_0$ (due to the decreased bubble volume). The new height $\tilde{w}$ can be found from an implicit relation $\tilde{w} = C \cdot (1 - \nu^2)\frac{(\tilde{p}_0 - p_\mathrm{e} - p_\mathrm{a}) \cdot b^4}{E \cdot d^3}$, where $\tilde{p}_0 = p_0 \frac{w-d}{\tilde{w}-d}$. Solving these two coupled equations we obtain the change in the height of the bubble $w - \tilde{w} \cong 200$ nm at an electrostatic pressure of 20 atm, which is comparable with the estimates we obtained from optical spectra fitting. It is necessary to stress that the estimate is quite rough due to the simplicity of the model and the possible presence of hydrocarbons and water inside the bubble.

Future nanomechanical modulators could benefit from decreasing the overall air gap thickness. Reducing the thickness of the air gap would also reduce the gate voltage required to produce the same Maxwell stress. Casimir forces could further enhance the modulation if the thickness of the hBN was decreased to bring the graphene-gold distance down to a few tens of nanometres[5]. In fact, RCWA simulations reveal that even the spectral positions of the Rayleigh resonances within our structure become increasingly sensitive to *d* as the air gap is reduced (Supplementary Figure 3). Reduction and optimisation of the structural dimensions of future devices could lead to further enhancement of modulation depths at all wavelengths.

Supplementary Note 4 | Frequency dependence of modulation

As mentioned in the main text, high speed experimental characterization of the device was beyond the scope of this work, particularly as the device was not optimized for high frequency operation (e.g. we used graphene as one of the contacts, the area size was not optimized, etc.). However, we did measure the optical response of our modulator in the frequency range of 100 Hz - 100 kHz available in our setup.

Supplementary Figure 7 shows two examples of the operation of our modulator in reflection at a frequency of 1 kHz and 4 MHz. The green oscilloscope trace shows the applied ac gating voltage (which was superimposed with an offset gating voltage) and the yellow trace shows the modulated optical signal measured with a photodiode.

The typical frequency dependence of the optical modulation amplitude measured with a lock-in amplifier is given in Supplementary Figure 8a. We can see that the device still modulates light at 100 kHz. Overall, it has an overdamped response (with damping frequency of 8 kHz) connected to the electrical part of our device (the inverse RC time constant for the gating voltage was estimated around 0.1 ms). The red curve of the plot in Supplementary Figure 8b shows the overdamped fit to the measured frequency dependence.

The fit is quite good except in the high frequency range approaching ~100 kHz where we see a shallow peak of the experimental response (which is most probably connected with the mechanical resonance). In order to describe the combined electro-mechanical properties of the device we have fitted the measured data as the product of a harmonic oscillator response and the overdamped electrical response (green line in Supplementary Figure 8b), which fits the experimental data extremely well. This fit provides a mechanical resonance frequency of ~120 kHz, which is in good agreement with estimates described in Supplementary Note 3.

Supplementary References

1 Griffiths, D. J. *Introduction to Electrodynamics*. 3rd edn (Pearson, 1999).
2 Kern, G., Kresse, G. & Hafner, J. Ab initio calculation of the lattice dynamics and phase diagram of boron nitride. *Phys. Rev. B* **59**, 8551-8559 (1999).
3 Inman, D. J. *Engineering Vibration*. 4th edn (Pearson, 2014).
4 Dennis, B. *et al.* Compact nanomechanical plasmonic phase modulators. *Nat. Photon.* **9**, 267-273 (2015).
5 Bordag, M., Fialkovsky, I., Gitman, D. & Vassilevich, D. Casimir interaction between a perfect conductor and graphene described by the Dirac model. *Phys. Rev. B* **80**, 245406 (2009).
